# Pubertal emergence of testosterone effects on depressive symptoms in boys

**DOI:** 10.1002/jcv2.12088

**Published:** 2022-07-13

**Authors:** Kristen M. Culbert, Antonio Milá Roa, Kimberly Stevens, Cheryl L. Sisk, S. Alexandra Burt, Kelly L. Klump

**Affiliations:** ^1^ Department of Psychology Michigan State University East Lansing Michigan USA; ^2^ Department of Psychology University of Nevada Las Vegas Nevada USA; ^3^ Neuroscience Program Michigan State University East Lansing Michigan USA

**Keywords:** depression, hormones, males, puberty, testosterone

## Abstract

**Background:**

Puberty‐driven increases in the secretion of testosterone may be a biological factor that protects males against the development of depression. Although all males produce testosterone, there are substantial between‐person differences that could contribute to differential vulnerability to depression among pre‐adolescent and adolescent boys, particularly after pubertal onset. Indeed, experimental animal and human data have shown that low testosterone increases risk for depressive‐like symptoms in males, whereas higher levels of testosterone may be protective; however, prior studies have primarily investigated these effects in adulthood. This study investigated whether lower circulating levels of testosterone predict depressive symptoms in pre‐adolescent and adolescent boys, and in particular, whether the testosterone‐depression association becomes prominent with advancing pubertal maturation.

**Methods:**

Male twins (*N* = 213; ages 10–15 years) from the Michigan State University Twin Registry self‐reported their depressive symptoms and pubertal status using the Children's Depression Inventory and the Pubertal Development Scale, respectively. Salivary testosterone was assayed using high‐sensitivity enzyme immunoassays. Mixed Linear Models (MLMs), which could account for the non‐independence of twin data, were used for analyses.

**Results:**

As expected, lower testosterone concentrations were associated with higher depressive symptoms, and the magnitude of this effect increased with advancing pubertal status. In contrast, boys with higher levels of testosterone showed low levels of depressive symptomatology at all stages of pubertal maturation.

**Conclusions:**

Overall, these findings enhance understanding of within‐sex variability in risk for depression in boys – average‐to‐high testosterone levels may underlie the general male resilience to depression after pubertal onset, whereas lower levels may increase vulnerability during/after puberty.

## Graphical abstract

This study aimed to determine whether the association between lower testosterone and depressive symptoms becomes prominent with advancing pubertal maturation. We found that depressives symptoms were higher in boys with lower testosterone concentrations, yet this effect was only evident in mid‐puberty and beyond. In contrast, higher levels of testosterone were associated with low symptom endorsement in all stages of puberty.

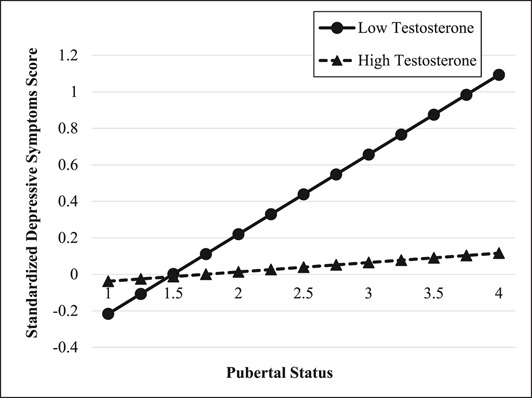

**Key points**

- Testosterone may impact differential risk for depression among boys and men. Low testosterone leads to the development of depression in adult men, but there is a dearth of research on pre‐adolescent and adolescent boys. Whether pubertal maturation moderates testosterone‐depression associations has also remained largely unexplored.
- This study investigated whether pubertal maturation contributes to the detection of a testosterone‐depression association. We aimed to determine whether the association between lower testosterone and depressive symptoms becomes prominent with advancing pubertal maturation.
- We found that depressives symptoms were higher in boys with lower testosterone concentrations, yet this effect was only evident in mid‐puberty and beyond. In contrast, higher levels of testosterone appeared to contribute to a more “male‐typical” pattern of low symptom endorsement – depressive symptoms remained blunted in boys in all stages of puberty.
- Results highlight puberty as a key developmental period for the emergence of testosterone‐depression associations. Studies that can provide further insight into *how* testosterone exerts its risk/protective effects within the CNS will be critical for enhancing etiologic models of depression in boys and for identifying novel targets for treatment.

## INTRODUCTION

Epidemiological data indicate that sex differences (i.e., higher rates in females than males) in the prevalence of clinical depression and depressive symptoms become evident around puberty (Frey et al., 2020; Salk et al., 2017). Average rates of depressive symptoms decrease or remain stable in boys after the onset of puberty, whereas increases are observed in females (e.g., Angold et al., 1998; Conley & Rudolph, 2009). This sex disparity has led to an abundance of research exploring elevated risk for depression in females, yet factors that may contribute to the reduced risk for depression in boys remain largely unknown. Testosterone is a prime candidate to consider given that testosterone influences sexual differentiation of the central nervous system (CNS; Juraska et al., 2013), rises in males during puberty, and impacts neural activity in affective and motivation/reward regions (e.g., hippocampus, amygdala, nucleus accumbens) implicated in depression (Wierenga et al., 2018; Williams et al., 2019). Furthermore, although all males produce testosterone, there are substantial between‐person differences in circulating levels which may map onto individual differences in risk for depression, particularly after pubertal onset.

To date, most animal and human studies exploring the link between testosterone and depression have focused on *adulthood*, rather than adolescence/puberty. For example, gonadectomy (i.e., removal of the gonadal source of circulating testosterone) in adult male rats increases depressive‐like features (Wainwright et al., 2011), and testosterone treatment in gonadally‐intact and gonadectomized adult male rats/mice results in reductions of depressive‐like symptoms, suggesting that testosterone may have “antidepressant” effects (e.g., Frye & Walf, 2009; Wainwright et al., 2016). Similarly, men with depressive symptoms have lower levels of testosterone than non‐depressed men (e.g., Barrett‐Connor et al., 1999; Fischer et al., 2019; McHenry et al., 2014; Shores et al., 2005), and low testosterone has also been correlated with depressive symptom severity (e.g., Almeida et al., 2008; Barrett‐Connor et al., 1999; Shores et al., 2005). Data also demonstrate that testosterone replacement therapy reduces depressive symptoms in hypogonadal men (e.g., McHenry et al., 2014; Zarrouf et al., 2009), including in men whose symptoms have not improved with the use of conventional antidepressant medication (e.g. SSRI's; Seidman et al., 2005). Animal and human data therefore converge in suggesting that testosterone may be protective against depressive symptoms in adult males.

Despite evidence of testosterone effects on depressive symptoms during adulthood, surprisingly few studies have examined the adolescent/pubertal period. Findings from the few studies that have been conducted generally indicate that boys with lower levels of circulating testosterone report higher levels of negative mood: internalizing symptoms (i.e., latent anxiety‐depression; Granger et al., 2003), sad affect (Susman et al., 1985, 1987, 1991), and depressive symptoms (Susman et al., 1991). Nonetheless, most of these published reports were conducted by a single research group using a small sample size (e.g., 56 boys; Susman et al., 1985, 1987, 1991) and some mixed findings emerged depending on the specific mood outcome or time point examined (e.g., time 1 vs. time 3 in longitudinal data; Susman et al., 1991). Furthermore, samples have been primarily comprised of boys in advanced pubertal stages (e.g., Granger et al., 2003; Susman et al., 1985), and when studies have considered a potential role for maturational status (e.g., age, pubertal development; Granger et al., 2003; Susman et al., 1987, 1991), they statistically controlled for between‐person variability rather than directly testing whether the testosterone‐depression association is *moderated* by pubertal status. Consequently, prior findings cannot speak to whether the effects of low testosterone on depressive symptoms in boys become pronounced with advancing pubertal maturation.

Exploring whether pubertal status moderates testosterone‐depression effects in boys is a critical next step. There are natural individual differences in circulating testosterone levels in all maturational stages, yet the relative impact of testosterone levels on depression could vary by pubertal status. Moreover, puberty is a key developmental period of neural organization (i.e., long‐lasting changes to the CNS) – a process that is driven by sex steroids and includes alterations of brain regions responsible for emotional processing (Juraska et al., 2013; Schulz & Sisk, 2016). Following pubertal neural organization, the CNS shows increased responsiveness to the activational (i.e., transient; based on the presence/level of the hormone) effects of circulating testosterone (Schulz & Sisk, 2016). Consequently, the degree of pubertal maturation may be important for detecting testosterone‐depression associations and critical for understanding when testosterone's risk/protective effects on depression become relevant in boys.

This study aimed to build upon prior findings by investigating whether individual differences in circulating levels of testosterone are associated with individual differences in depressive symptoms, and in particular, whether the testosterone‐depression association becomes prominent with advancing pubertal maturation. Moreover, we tested two plausible yet competing hypotheses. We first examined a main effect of testosterone on depressive symptoms, adjusting for age and pubertal status, as has been explored in prior research (e.g., Granger et al., 2003; Susman et al., 1987) since this would test whether higher testosterone is associated with lower levels of depressive symptoms (and vice versa), irrespective of maturational status. We then tested for potential interactive effects between testosterone and pubertal status to determine whether testosterone's “protective effects” on depressive symptoms in boys become evident with advancing pubertal maturation. If only a main effect of testosterone on depressive symptoms is important, then pubertal development could be linked to low depression risk in boys merely because pubertal status serves as an indirect proxy for the presence of circulating testosterone and its biological protective effects. In contrast, if pubertal status moderates the testosterone‐depression association, then pubertal maturation may have etiologic relevance and could be indicative of developmental shifts in the CNS's responsiveness to the risk/protective effects of circulating testosterone.

In addition to our primary aims/analyses, a series of post hoc analyses were conducted to further elucidate testosterone‐depression relationships in boys during puberty. Our first set of post hoc analyses aimed to determine the specificity of our findings in relation to other psychopathological outcomes and to evaluate whether a stressor, that is, parent‐child conflict, might influence our results. Anxiety and aggression were tested as additional psychopathological outcomes as well as covariates since these symptoms/behaviors often co‐occur with depression in boys (Essau & Chang, 2009) and have been linked to testosterone in some prior work (Duke et al., 2014). Parent‐child conflict was tested as a potential contextual modifier and covariate since prior data has shown that lower parent‐child relationship quality can exacerbate the strength of the association between lower testosterone and depressive symptoms in pubertal boys (Booth et al., 2003). Finally, because steroid hormones, like testosterone, regulate gene transcription (e.g., activation/inhibition of gene expression) in the CNS (McEwen, 1994), a second set of post hoc analyses used intraclass twin correlations to tentatively explore whether lower levels of testosterone may influence depressive symptoms in boys via testosterone's modulation of genetic effects.

## METHODS

### Participants

This study was approved by the Michigan State University (MSU) Institutional Review Board and informed consent/assent was appropriately obtained. Participants were male twins (*N* = 213) ages 10–15 years (*M*_age_ = 12.74, SD = 1.62) who had completed the MSU Twin Registry (MSUTR) “Adolescent Twin Hormone Study” (conducted 2006–2009; see Burt & Klump, 2013, 2019). Twin status was not central to our primary research question; this dataset was used because it contained the necessary variables to test phenotypic associations between testosterone and depressive symptoms in boys of varying pubertal status. Given that our primary analyses focused on phenotypic effects, we could include all male twins from the parent study irrespective of their twin type. The total sample was comprised of 86 boys from same‐sex monozygotic twin pairs, 62 boys from same‐sex dizygotic twin pairs, and 65 boys from opposite‐sex dizygotic twin pairs.

The MSUTR collaborates with the Michigan Department of Health and Human Services to recruit twins within the targeted age‐range and recruitment region (e.g., ∼2 h radius from MSU) through the use of birth records. Because birth records are confidential, recruitment packets are mailed directly from the MDHHS to eligible twin‐pairs. Mailings are repeated until a response is received or four mailings have been sent (Burt & Klump, 2019). In this study, families mailed back a pre‐stamped postcard with their contact information or directly contacted the MSUTR project office. Study staff conducted phone screenings to determine study eligibility and schedule their assessment. The recruitment response rate (56%) was on par with those of other population‐based twin registries that used similar approaches (Burt & Klump, 2013).

Exclusion criteria were medication use (e.g., anti‐inflammatory steroid) or medical conditions (e.g., diabetes) that could alter hormone function; there were no other exclusion criteria. The single‐day assessment involved one parent (who completed questionnaires on themselves as well as questionnaires on each twin) and their twins (who provided saliva samples and completed self‐report questionnaires). Families were compensated up to $150 for their participation. Importantly, twins are representative of the general population on several measures of behavior and development (e.g., Barnes & Boutwell, 2013), and race/ethnicity endorsements of participating families were comparable to the local census (Burt & Klump, 2013). Most boys were White (*n* = 188; 88.3%) and the remaining were Black (*n* = 14; 6.6%), Asian/Pacific Rim (*n* = 2; 0.9%), American Indian (*n* = 1; 0.5%), or multi‐racial (*n* = 8; 3.8%); a proportion of these boys were of Hispanic/Latinx origin (*n* = 7; 3.3%).

### Measures

#### Depressive symptoms

The Children's Depression Inventory (CDI; Kovacs, 1985) assessed depressive symptoms over the past 2 weeks, in terms of negative mood (e.g., feeling sad), anhedonia (e.g., impairments in experiencing pleasure), interpersonal problems (e.g., difficulties interacting with others, social avoidance/isolation), ineffectiveness (e.g., negative perception of one's ability/performance), and negative self‐esteem (e.g., low self‐esteem). Notably, a suicidal ideation item was not administered since it was a non‐clinical sample and rates of suicidality would be expected to be low (Kokkevi et al., 2012); the total score therefore reflects the sum of all other items. Higher scores reflect higher depressive symptomatology.

The CDI has been validated for use in children as young as 7 years old and has shown good psychometrics for males in previous studies (Figueras Masip et al., 2010; Kovacs, 1985). The CDI total score has also been found to discriminate between depressed and nondepressed adolescents (e.g., Figueras Masip et al., 2010). We focused on the CDI total score in this study since internal consistency was adequate (*α* = .82). Lower internal consistency was found on subscales (*α* = .47–.69).

#### Pubertal status

Pubertal status was evaluated using the male version of the Pubertal Development Scale (PDS; Petersen et al., 1988), which assesses a wide range of male secondary sex characteristics (e.g., voice changes, appearance of body/facial hair, skin changes). Items are rated on a continuous 4‐point scale ranging from (1) development has not yet begun to (4) development seems complete. We used the continuous total score (i.e., average PDS score) in our analyses to estimate testosterone‐depression associations dimensionally, across the full range of puberal maturation (i.e., from pre‐puberty to post‐puberty). Higher scores are indicative of more advanced pubertal development. The PDS shows good psychometrics in boys (Culbert et al., 2017; Petersen et al., 1988), including high correlations between self‐report PDS ratings and clinician‐reports (*r* ∼ 0.70; Petersen et al., 1988). Internal consistency was adequate (*α* = 0.86) in our sample, and self‐reported ratings correlated highly with parent ratings (*r* = 0.82).

#### Testosterone

Passive drool saliva samples (4 ml) were collected from boys between 13:00 and 16:45 (*M* = 14:07, SD = 0:54) and were assayed in duplicate using high‐sensitivity enzyme immunoassays to determine testosterone concentrations. Saliva was considered advantageous over other biomarkers, like serum or plasma, since it is less invasive and salivary testosterone levels correlate very highly with those from serum (*r* = 0.91–0.96; Granger et al., 1999). Additionally, testosterone concentrations from saliva reflect the unbound, or biologically active, fraction of this steroid (Granger et al., 1999).

Participants were asked to fast for 4 h prior to saliva collection and to not brush their teeth for at least 30 min prior to collection. When screened for compliance, most participants (92.5%) reported that they had refrained from eating/drinking in the past 4 h, and all had refrained from brushing their teeth. Collection occurred in the late afternoon since diurnal variation in testosterone is most minimal at this time of day (Granger et al., 1999). Following collection, samples were immediately placed in a −20°C freezer until they were shipped to Salimetrics, LLC (State College, Pennsylvania, USA) for testing, using the Salimetrics Testosterone Enzyme Immunoassay Kit. On the day of testing, samples were centrifuged at 3000 revolutions per minute for 15 min to remove mucins. Clear samples were transferred into testing wells to screen for potential problems with pH. Samples that fell out of the pH range of 4–9 were diluted in phosphate‐buffered saline to correct pH prior to testing. The testosterone assay had a minimum detection limit of 1 pg/ml and average intra‐ and inter‐assay coefficients of variations that were less than 4.60% and 8.25%, respectively. Method accuracy, determined by spike recovery and linearity, was within acceptable ranges (104.4% and 99.9%), and all boys had testosterone concentrations above the minimum detection limit.

##### Post hoc variables

Variables (i.e., anxiety, aggression, and parent‐child conflict) included in post hoc analyses were assessed using well‐validated measures: Multidimensional Anxiety Scale for Children (MASC; March et al., 1997 in Supporting Information S1), Early Adolescent Temperament Questionnaire‐Revised (EATQ‐R; Capaldi & Rothbart, 1992; Ellis & Rothbart, 2001 in Supporting Information S1), and Parental Environment Questionnaire (PEQ; Elkins et al., 1997 in Supporting Information S1). For measurement details, please refer to our Supporting Information S1.

## STATISTICAL ANALYSES

Analyses were conducted with IBM SPSS Statistical Software for Windows, version 28.

### Data preparation

Body Mass Index (BMI) was calculated using height and weight measurements collected in the laboratory; weight in kilograms was divided by height in meters squared, adjusting for age and sex, using the Center for Disease Control BMI‐for‐age calculator: https://www.cdc.gov/healthyweight/xls/bmi_group_calculator_metric.xls. Depressive symptom scores were prorated to adjust for the exclusion of the CDI suicidality item, but there were no other missing data for study variables. Testosterone, depressive symptoms, and BMI were rank‐ordered and Blom‐transformed prior to analyses to adjust for slight positive skewness (1.14–1.93). Following transformation, skewness values were closer to zero (0.002–0.130). Continuous variables were standardized prior to analyses to ease interpretation of model coefficients.

### Statistical models

Mixed linear models (MLMs) were used to control for the non‐independence of twin data, that is, the level‐1 variable (individual twin) was nested within the level‐2 unit (twin pair). The first set of MLMs focused on evaluating whether there is a significant main effect of testosterone on depressive symptoms, adjusting for individual variation in pubertal status. The second set of MLMs focused on evaluating the potential interplay between testosterone and pubertal status; thus, we examined the main effect of testosterone, main effect of pubertal status, and the testosterone x pubertal status interaction on depressive symptoms. A significant interaction would suggest that testosterone's association with depressive symptoms varies by pubertal maturation, and follow‐up with simple slopes analysis would be used to determine the effect of testosterone on depressive symptoms at different levels of pubertal status.

After fitting the “initial” main effect and interaction models, “covariate” models were tested. Covariate models adjusted for age, BMI, saliva collection time, and fasting status to ensure that these potential confounds did not substantially alter the findings. Age and BMI were included as covariates given their associations with testosterone, puberty, and depressive symptoms (e.g., Granger et al., 2003; Mogri et al., 2013). Saliva collection time and fasting status were included as covariates given between‐subject variation in the timeframe of collection and the fact that a subset of participants (7.5%) reported food/beverage consumption within 4 h prior to the assessment, which can impact testosterone levels (Terrier & Isidori, 2016).

#### Post hoc analyses

Anxiety symptoms, aggression, and parent‐child conflict were included as additional covariates in main effect and interaction MLMs to confirm that adjusting for these post hoc variables did not unduly influence results. Additionally, anxiety symptoms or aggressive behavior were entered as outcomes and then similar MLMs (main effect and interaction models, as described for depressive symptoms) were used to test the main effect of pubertal status, main effect of testosterone, and the testosterone × pubertal status effect on these symptoms/behaviors. To evaluate parent‐child conflict as a possible contextual modifier, a post hoc MLM estimated two‐way and three‐way interactions between testosterone, pubertal status, and parent‐child conflict on depressive symptoms.

Post hoc intraclass twin correlations were conducted to tentatively explore whether genetic influences on depressive symptoms may vary as a function of circulating levels of testosterone. Monozygotic (MZ) twins are genetically identical, whereas dizygotic (DZ) twins share ∼50% of their genes; thus, comparisons between monozygotic (MZ) and dizygotic (DZ) twin correlations can help disentangle genetic and environmental influences on an outcome, like depressive symptoms. In these post hoc analyses, same‐sex MZ and DZ male twins were separated into low versus high testosterone groups using the median split for testosterone levels (41.18 pg/ml) and then intraclass rMZ versus rDZ correlations were compared in each testosterone group. When the rMZ correlation is at least twice as similar as the rDZ correlation, results are suggestive of genetic effects. Additional methodological details are provided in Supporting Information (see Figure S1).

## RESULTS

### Descriptive statistics

Testosterone and depressive symptoms showed adequate variability (see *M*, SD, range in Table 1). A total of 3.3% (*n* = 7) of boys scored above the recommended CDI clinical cutoff for non‐clinical/community samples (score = 19), which is consistent with population prevalence estimates of depression in this age range (3.2%; Ghandour et al., 2019). As expected, salivary testosterone and pubertal status were highly correlated (*r* = 0.69, *p* < 0.001), but were not interchangeable; each variable also captured a substantial proportion of unique variance (i.e., shared variance: *r* ^2^ = 0.47). Additionally, small non‐significant associations were found between pubertal status and depressive symptoms as well as testosterone and depressive symptoms (Table 1). The negligible link between pubertal status and depressive symptoms is consistent with prior research that has found relatively similar levels of depression in boys after the onset of puberty.

**TABLE 1:** Bivariate Pearson correlations and descriptive statistics (*N* = 213)

						Post‐hoc psychological variables		
	Testosterone	Pubertal status	Depressive symptoms	Age	BMI	Anxiety	Aggression	Parent–child conflict
Bivariate correlations								
Testosterone	–	0.69**	−0.03	0.64**	0.38**	−0.22**	−0.06	−0.04
Pubertal status		–	0.05	0.72**	0.39**	−0.25**	0.08	−0.04
Depressive symptoms			–	−0.02	0.13	0.35**	0.49**	0.46**
Age				–	0.36**	−0.24**	−0.02	−0.09
BMI					–	−0.17*	−0.01	0.01
Post hoc variables								
Anxiety						–	0.22**	0.02
Aggression							–	0.36**
Parent‐child conflict								–
Descriptive data								
Full sample								
Maximum score	–	4.00	52.00	–	–	117.00	30.00	48.00
Range	9.59–183.05	1.00–4.00	0.00–36.35	10.02–15.77	13.60–34.90	0.00–117.00	6.00–28.00	12.00–48.00
Mean (SD)	52.98 (34.24)	1.93 (0.78)	6.19 (5.39)	12.74 (1.62)	19.82 (3.87)	46.90 (16.51)	13.78 (4.31)	22.99 (6.89)

*Note*: Bold text indicates significant correlations.

Abbreviation: BMI, Body Mass Index.

*p ≤ .01; **p < .001.

### Primary MLM analyses

**FIGURE 1:**
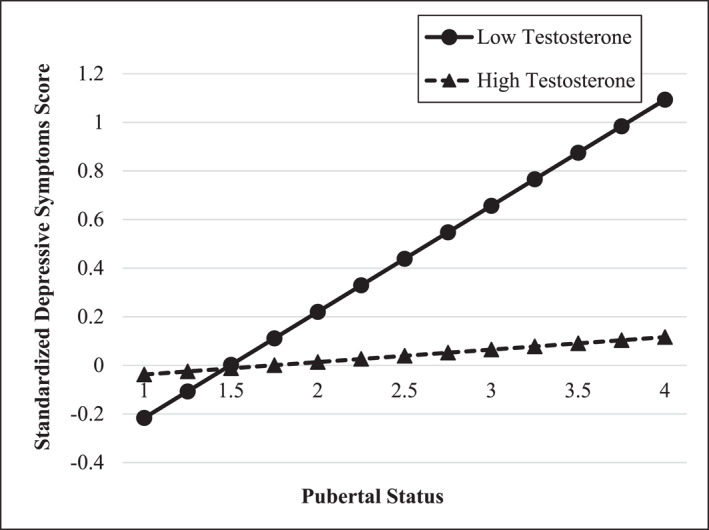
Individual differences in testosterone levels predict individual differences in depressive symptoms in boys during puberty. Plotted results reflect the covariate interaction model (adjusted for age, BMI, saliva collection time, and fasting status). The *y*‐axis reflects the predicted (standardized) depressive symptoms scores, and (standardized) testosterone levels were plotted according to 1 SD. below the mean (“low testosterone levels” which corresponded to an untransformed raw value of ∼23 pg/ml) and 1 SD above the mean (“high testosterone levels” which corresponded to an untransformed raw value of ∼88 pg/ml). Although standardized pubertal status scores were used in analyses, raw Pubertal Development Scale values are depicted on the *x*‐axis to ease interpretation

There was no significant association between testosterone and depressive symptoms in MLM main effect models (Table 2). MLM interaction models revealed a significant testosterone x pubertal status effect on depressive symptoms, which remained even after adjusting for all covariates (Table 2). Specifically, lower levels of testosterone predicted higher levels of depressive symptoms, but the magnitude of this association became prominent with advancing pubertal development (Figure 1). Follow‐up simple‐slopes tests indicated that lower testosterone was associated with increased depressive symptoms in boys who were in mid‐to‐late puberty (mid‐puberty: *β* = −0.20, *p* < 0.05; late puberty: *β* = −0.47, *p* = 0.01), whereas no significant association between testosterone and depressive symptoms was present in boys who were in pre‐to‐early puberty (pre‐puberty: *β* = 0.09, *p* = 0.48; early puberty: *β* = −0.06, *p* = 0.57).^1^

**TABLE 2:** Testosterone and pubertal status effects on depressive symptoms in boys (*N* = 213)

Depressive symptoms	Initial models		Covariate models		Post‐hoc covariate models	
	Coefficient (S.E.)	*p*	Coefficient (S.E.)	*p*	Coefficient (S.E.)	*p*
Main effect models						
Testosterone	−0.11 (0.09)	0.24	−0.11 (0.09)	0.26	−0.06 (0.07)	0.41
Covariates						
Pubertal status	0.12 (0.09)	0.17	0.14 (0.10)	0.19	0.07 (0.08)	0.40
Age	–	–	−0.10 (0.10)	0.34	0.03 (0.08)	0.76
BMI	–	–	0.14 (0.07)	0.06	**0.14** (**0.06**)	**0.02**
Saliva collection time	–	–	−0.02 (0.07)	0.69	−0.01 (0.05)	0.87
Fasting status	–	–	0.21 (0.25)	0.41	0.24 (0.20)	0.21
Post‐hoc additional covariates						
Anxiety	–	–	–	–	**0.30** (**0.06**)	**<0.001**
Aggression	–	–	–	–	**0.28** (**0.06**)	**<0.001**
Parent–child conflict	–	–	–	–	**0.35** (**0.06**)	**<0.001**
Interaction Models						
Testosterone	−0.09 (0.09)	0.29	−0.09 (0.09)	0.33	−0.05 (0.07)	0.47
Pubertal status	0.17 (0.09)	0.06	0.19 (0.11)	0.08	0.11 (0.08)	0.20
Testosterone × pubertal status	**−0.15** (**0.06**)	**0.02**	**−0.15** (**0.06**)	**0.02**	**−0.12** (**0.05**)	**0.03**
Covariates						
Age	–	–	−0.10 (0.10)	0.31	0.02 (0.08)	0.83
BMI	–	–	0.14 (0.07)	0.07	**0.14** (**0.06**)	**0.02**
Saliva collection time	–	–	−0.04 (0.07)	0.57	−0.02 (0.05)	0.68
Fasting status	–	–	0.21 (0.25)	0.41	0.25 (0.19)	0.20
Post‐Hoc Additional Covariates						
Anxiety	–		–		**0.29** (**0.05**)	**<0.001**
Aggression	–		–		**0.27** (**0.06**)	**<0.001**
Parent‐child conflict	–		–		**0.35** (**0.06**)	**<0.001**

*Note*: Continuous variables were standardized prior to analysis so coefficients reflect standardized effects. Fasting status was coded 0 (fasted for 4 h) or 1 (did not fast). The Post‐Hoc Covariate Models included all of the initial study covariates (e.g., age, BMI, Saliva Collection Time), plus three additional covariates: anxiety, aggression, and parent‐child conflict. Bold text indicates significant effects.

Abbreviation: BMI, Body Mass Index.

### Post hoc analyses

As noted previously, post hoc analyses were used to explore the specificity of our findings in relation to other psychopathological outcomes (i.e., anxiety symptoms and aggressive behavior) and to evaluate whether parent‐child conflict might be a contextual modifier that impacts the relationship between testosterone and depressive symptom in boys during puberty. Our primary MLM results (i.e., testosterone × pubertal status interaction effect on depressive symptoms) remained significant even when anxiety symptoms, aggressive behavior, and parent‐child conflict were included as additional post hoc covariates (Table 2). Post hoc analyses also revealed that the testosterone‐pubertal status interplay does not extend to anxiety symptoms or aggression; a significant main effect of pubertal status was found for these outcomes but estimates for testosterone and the testosterone‐pubertal status interaction were non‐significant in these analyses (see Supporting Information, Table S1). Finally, although higher levels of parent‐child conflict were associated with higher levels of depressive symptoms, parent‐child conflict did not significantly interact with pubertal status, testosterone, or pubertal status × testosterone effects on depressive symptoms in boys (see Supporting Information, Table S2).

Additionally, post hoc twin intraclass correlations were conducted to get an initial indication of whether lower levels of testosterone may enhance genetic influences on depressive symptoms in boys during puberty (see Supporting Information, Figure S1). The intraclass correlation for depressive symptoms was higher in identical (i.e., monozygotic (MZ)) than in fraternal (i.e., dizygotic (DZ)) male twin pairs, especially in the low testosterone group – a pattern that aligns with the possibility that testosterone alters risk for depressive symptoms via genomic effects.

## DISCUSSION AND CONCLUSION

This study investigated whether pubertal status is important for understanding testosterone's effects on depressive symptoms in boys. We found no evidence of a main effect of testosterone; instead, testosterone interacted with pubertal status to predict differential levels of depressive symptoms. Boys with lower testosterone endorsed significantly higher levels of depressive symptoms and this effect became evident after pubertal onset, particularly mid‐puberty and beyond. Conversely, boys with higher testosterone reported lower depressive symptoms at all levels of pubertal maturation. Results were specific to depressive symptoms (as opposed to anxiety or aggressive behavior) and remained unchanged even after adjusting for several potential confounds (e.g., BMI, fasting status, anxiety, aggression, and conflictual parent‐child relationships). Taken together, boys who produce naturally lower testosterone may be most vulnerable to developing depression during/after puberty, whereas average‐to‐high testosterone concentrations may be protective.

Importantly, findings converge with animal and human data *in adulthood* that have shown that low testosterone increases risk for depressive symptomatology and build upon prior evidence of associations between lower testosterone and negative mood in boys at *advanced stages of puberty* (e.g., Granger et al., 2003; Zarrouf et al., 2009). In particular, our data highlight the critical importance of considering maturational level when exploring testosterone‐depression associations. While directionality and causation cannot be determined from our cross‐sectional data, we posit that there may be something about pubertal development that exacerbates the relationship between low testosterone and depressive symptoms; a hormonal milieu of relatively low testosterone at earlier points of development (e.g., during pre‐to‐early puberty) does not appear to be biologically “risky.” The mechanisms underlying this developmental pattern remains to be elucidated, but testosterone‐dependent organization and activation of the CNS during puberty could be at play.

Prenatal/perinatal development is the first critical period for testosterone‐dependent organization and masculinization of the CNS in males, but a second bout of testosterone‐driven neural (re)organization/masculinization occurs during puberty (Schulz & Sisk, 2016). For example, exogenous administration of testosterone cannot activate adult male‐typical phenotypes (e.g., sexual behavior) in male rodents prior to puberty, and male‐typical behaviors are also not activated by testosterone after puberty if rodents are not exposed to sufficient testosterone during the pubertal period (e.g., Schulz & Sisk, 2016). If we apply this pubertal organizational‐activational framework to depression, exposure to sufficient levels of pubertal testosterone (e.g., presumably average‐to‐high concentrations) would be expected to first organize neural circuits and then facilitate the male‐typical pattern of reduced depression risk via activation of those circuits. An insufficient level of pubertal testosterone (e.g., lower concentrations) might fail to facilitate these protective effects within the CNS, resulting in heightened depression risk. Our results align with this framework since activational effects alone (i.e., non‐significant main effect of testosterone) do not appear to be enough to facilitate individual differences in depressive symptoms in pre‐adolescent and adolescent boys; lower levels of testosterone only emerged as “risky” for depressive symptoms in the presence of advancing pubertal development. Moving forward, it will be critical to further explore organizational‐activational effects of testosterone on depressive symptoms in males during/after puberty via direct manipulation of testosterone (e.g., animal models of depression) and multi‐method longitudinal studies that incorporate hormonal, brain, and symptom assessments.

Future studies will also need to elucidate *how* lower testosterone enhances vulnerability to depressive symptoms in pubertal boys. As noted previously, a primary function of testosterone is genomic regulation within the CNS which shapes neural and behavioral outcomes (McEwen, 1994). Testosterone enters cells, binds to their androgen receptors, and acts on target genes (i.e., activates or inhibits gene transcription), including within mood and motivational/reward systems relevant to depression (e.g., serotonin; dopamine; McEwen, 1994). Exposure to sufficient levels of testosterone could regulate genetic risk (e.g., activate protective genomic effects; inhibit expression of risk genes) in a manner that reduces vulnerability to depression, whereas low testosterone levels could enhance risk (e.g., fail to inhibit risk genes). The current study could not use advanced behavior genetic models (e.g., twin moderation models) to test potential genetic effects since these techniques require several hundred twin pairs for adequate statistical power (Purcell, 2002), but results from twin intraclass correlations (see post hoc analyses; Figure S1) provide tentative evidence that lower levels of testosterone may enhance genetic influences on depressive symptoms in boys during puberty. Nonetheless, it will be critical for future studies to replicate these findings using much larger samples sizes and more advanced genetically informed approaches.

Additionally, an interplay of biopsychosocial mechanisms could influence the relation between testosterone and depressive symptoms. Negative life events and heightened levels of stress are often involved in the onset of mood syndromes (Roy & Campbell, 2013), and contextual factors have been found to enhance or attenuate testosterone associations with psychological outcomes (Duke et al., 2014). Although parent‐child conflict was not a significant contextual modifier in our data, this does not negate the fact that other risk factors (e.g., critical life events; peer rejection; Conley & Rudolph, 2009) could be at play. Furthermore, at a biological level, there may be important interconnections between stress, testosterone, and negative mood that could impact individual variability in risk for depression in boys. Chronic stress can lead to glucocorticoid over‐production which blunts testosterone secretion and also alters mood‐related systems (e.g., serotonin neurotransmission; Roy & Campbell, 2013; Tafet & Bernardini, 2003). Extant animal data have also shown that stress responsiveness is higher during puberty/adolescence than other stages of development (e.g., adulthood), and importantly, testosterone naturally reduces stress reactivity in males (Romeo et al., 2016; Williams et al., 2019) – an effect that emerges after pubertal onset (Romeo et al., 2004) and is linked to testosterone's neural modulation of stress/emotion and motivation/reward circuits (Tafet & Bernardini, 2003; Williams et al., 2019). Thus, higher levels of testosterone might enhance male resilience to depression, during and after puberty, by reducing stress reactivity and buffering against the negative impact of contextual risk factors/stressors. Developmental human studies that explore these intersecting biopsychosocial effects are needed.

Findings from this study are important, but limitations must be noted. We tested testosterone concentrations from saliva samples, rather than blood. Salivary testosterone concentrations are highly correlated with those obtained from blood (Granger et al., 1999); however, the replication of our finding using blood samples is also needed. Additionally, although salivary samples were tested in duplicate, samples were collected on a single day and only in the afternoon, and other hormones (e.g., estrogen, cortisol) that influence psychological symptoms and can act jointly with testosterone were not tested (Cobb et al., 2018; Fischer et al., 2019). Future studies should examine hormone‐symptom effects across multiple days/timepoints to determine the stability of effects and to confirm that within‐person differences in testosterone levels predict within‐person differences in depression across pubertal maturation. Exploration of other hormones and their potential interplay with testosterone effects is also warranted. Furthermore, we relied on self‐reports of compliance with fasting procedures, and it is possible that some self‐reports did not reflect actual adherence. Participants were also not screened for nicotine, alcohol, or other drug use (e.g., cannabis, opioids) that can alter testosterone production (Duca et al., 2019); thus, substance use screening could be important for future work. We also assessed depressive symptoms in a community sample using a single, but well‐validated, self‐report measure. The inclusion of additional measures of depression would have been beneficial. Moreover, although endorsement of depressive symptoms spanned a spectrum of severity, the extent to which lower testosterone is associated with clinical depression in boys remains to be established. Future studies should incorporate interview‐based measures that can more definitively screen for clinical depression. Additionally, our (post hoc) results point to depression‐specific effects, at least in relation to anxiety symptoms or aggression, but it will be important to replicate these findings and to evaluate other disorders associated with testosterone and frequently comorbid with depression (e.g., Attention Deficit Hyperactivity Disorder; Substance Use; Essau & Chang, 2009; Duke et al., 2014). Finally, in terms of representativeness, our sample matched the racial/ethnic composition of the recruitment region, and twins are similar to non‐twins on measures of behavior and development, including depressive symptoms (e.g., Barnes & Boutwell, 2013; Burt & Klump, 2013; Burt & Klump, 2019). It is necessary, however, to test whether our findings extend to other samples, including samples that are more racially/ethnically diverse and samples that include other sexes and gender identities.

This study adds to the growing literature documenting sex steroid influences on depressive symptoms across the lifespan and especially during/after puberty. Based on our findings, puberty and natural between‐person variation in testosterone appear to be important for understanding individual differences in depressive symptoms among pre‐adolescent and adolescent boys. We suspect that pubertal maturation may play a critical role in facilitating CNS responsiveness to testosterone concentrations and its effects on depression in boys, but future studies are needed to further explore this possibility and elucidate the specific etiologic action(s) of testosterone within the CNS (e.g., genomic effects on neurotransmitter production; modification of neural circuitry or activation). Uncovering the specific mechanisms would advance etiologic models of depression in boys and could also result in the development of novel and more fine‐tuned pharmacological approaches for treatment.

## AUTHOR CONTRIBUTIONS

**Kristen M. Culbert:** Conceptualization (lead); Writing – original draft and review/editing (lead); Methodology (lead); Formal analysis and Visualization (lead); Funding Acquisition (equal); **Antonio Milá Roa:** Conceptualization (supporting); Writing original draft (supporting); Writing – review and editing (equal); Formal analysis (supporting); Funding Acquisition (equal). **Kimberly Stevens:** Writing – review and editing (equal); Formal analysis (supporting). **Cheryl L. Sisk:** Conceptualization (supporting); Writing – review and editing (equal); Funding Acquisition (equal). **S. Alexandra Burt:** Conceptualization (supporting); Project Administration and Resources (equal); Writing – review and editing (equal); Funding Acquisition (equal). **Kelly L. Klump:** Conceptualization (supporting); Project Administration and Resources (equal); Writing – review and editing (equal); Funding Acquisition (equal).

## CONFLICTS OF INTEREST

The authors have declared that they have no competing or potential conflicts of interest.

## ETHICAL CONSIDERATIONS

This study was approved by the Michigan State University (MSU) Institutional Review Board and participant informed consent/assent was appropriately obtained.

## Supporting information

Supporting Information S1

## Data Availability

The data that support the findings of this study are available on request from the corresponding author.

## ENDNOTES

^1^
The reported simple slope estimates were calculated based on the following pubertal status values: pre‐puberty (PDS score = 1; converted z‐score: −1.19473), early puberty (PDS score = 1.75; converted z‐score = −0.23202), mid‐puberty (PDS score = 2.50; converted z‐score: 0.7307), late puberty (PDS score = 3.75, converted z‐score = 2.33522). Although only four estimates are presented, we confirmed that simple slope tests were significant (*p*s < .05) for all PDS scores ≥2.5 (mid‐puberty to post‐puberty) and non‐significant (*p*s = .11‐.91) for all PDS scores <2.5 (pre‐puberty and early puberty; data available upon request).
