# Supplementary material for: Pubertal emergence of testosterone effects on depressive symptoms in boys
Source: JCPP Adv. 2022 Jul 13;2(3):e12088. doi: 10.1002/jcv2.12088 (PMC10195044; doi:10.1002/jcv2.12088)
Supplement: Supplementary file 1 — Supporting Information S1 [file JCV2-2-e12088-s001.docx]

**Supporting Information**

**METHODS**

**Post Hoc Measures**

Well-validated scales were used to assess post hoc variables (i.e., anxiety, aggression, and parent-child conflict), as detailed below.

**Anxiety Symptoms:** The total score from the Multidimensional Anxiety Scale for Children (MASC; March et al., 1997) was used to assess overall levels of anxiety symptoms (e.g., panic, social anxiety, physical symptoms). Participants rate how true each descriptive statement is for them on a 4-point scale, ranging from “never true about me” (0) to “often true about me” (3). Higher scores reflect higher levels of anxiety symptoms. The MASC has been validated for use in children as young as age 8 and has shown good psychometrics for pre-adolescent/adolescent males in prior work (e.g., March et al., 1997; March, Sullivan, Parker, 1999). MASC scores have also been found to discriminate between youth with and without anxiety disorders (e.g., Grills-Taquechel, Ollendick, & Fisak, 2008; Wood, Piacentini, Bergman, McCracken, & Barrios, 2002). Internal consistency was also good in our sample (α = 0.91).

**Aggression:** Early Adolescent Temperament Questionnaire-Revised (EATQ-R; Capaldi & Rothbart, 1992; Ellis & Rothbart, 2001) was used to assess aggressive behavior (e.g., physical violence, hostile reactivity, and verbal aggression). Participants rate how true each descriptive statement is for them on a 5-point scale, ranging from “almost always untrue” (1) to “almost always true” (5). Thus, higher scores reflect more aggressive behavior tendencies. The EATQ-R has been validated for use in children as young as age 9 and has shown good psychometrics in prior research (Capaldi & Rothbart, 1992; Ellis & Rothbart, 2001). Internal consistency was also acceptable in our sample (α = 0.75).

**Parent-child Conflict:** Parent-child conflict (e.g., disagreement, tension, anger in the parent-child relationship) was assessed with the Parental Environment Questionnaire (PEQ; Elkins et al., 1997). Each child reported on conflict with their father and conflict with their mother, and reports on both parents were available from 99.5% of the sample. Each item was rated on a 4-point scale from “definitely false” (1) to “definitely true” (4), and higher scores correspond to higher levels of parent-child conflict. The PEQ has shown good psychometric properties in prior studies of youth (e.g., Elkins et al., 1997; Klahr, Rueter, McGue, Iacono, & Burt, 2011), and internal consistencies were also acceptable in our sample (PEQ on mother: α = 0.88; PEQ on father: α = .91). Consistent with prior research (e.g., Burt, Clark, Gershoff, Klump, & Hyde, 2021; Klahr et al., 2011), a composite parent-conflict score was created (average score of adolescent report of conflict with their father and mother) and used in analyses to minimize the number of analyses conducted, particularly since reports on conflict with each parent were highly correlated (r = .67, *p* < .001) and the composite parent-conflict score showed the strongest association with depressive symptoms (r = .46, *p* <.001).

**Table S1.** Post Hoc Analyses Exploring Testosterone and Pubertal Status Effects on Anxiety Symptoms and Aggressive Behavior in Boys (N = 213).

|  | | ***Effects on Anxiety Symptoms*** | | | | |  | ***Effects on Aggressive Behavior*** | | | | |
| --- | --- | --- | --- | --- | --- | --- | --- | --- | --- | --- | --- | --- |
|  | | **Initial Models** | |  | **Covariate Models** | |  | **Initial Models** | |  | **Covariate Models** | |
|  |  | Coefficient (S.E.) | *p* |  | Coefficient (S.E.) | *p* |  | Coefficient (S.E.) | *p* |  | Coefficient (S.E.) | *p* |
| ***Main Effect Models*** |  | |  |  |  |  |  |  |  |  |  |  |
| Testosterone | | -0.11 (0.09) | .21 |  | -0.01 (0.09) | .95 |  | -0.17 (0.09) | .06 |  | -0.09 (0.08) | .30 |
| *Covariates:* | |  |  |  |  |  |  |  |  |  |  |  |
| Pubertal Status | | **-0.18 (0.09)** | **<.05** |  | -0.19 (0.10) | .06 |  | **0.19 (0.09)** | **.03** |  | **0.23 (0.10)** | **<.02** |
| Age | | -- | -- |  | -0.06 (0.10) | .55 |  | -- | -- |  | -0.06 (0.09) | .50 |
| BMI | | -- | -- |  | -0.13 (0.07) | .07 |  | -- | -- |  | -0.06 (0.06) | .34 |
| Saliva Collection Time | | -- | -- |  | -0.01 (0.06) | .87 |  | -- | -- |  | -0.10 (0.06) | .09 |
| Fasting Status | | -- | -- |  | -0.19 (0.23) | .42 |  | -- | -- |  | 0.23 (0.22) | .29 |
| Aggressive Behavior | | -- | -- |  | 0.10 (0.07) | .16 |  | -- | -- |  | -- | -- |
| Anxiety Symptoms | | -- | -- |  | **--** | **--** |  |  |  |  | 0.07 (0.08) | .28 |
| Depressive Symptoms | | -- | -- |  | **0.44 (0.08)** | **<.001** |  | -- | -- |  | **0.38 (0.08)** | **<.001** |
| Parent-Child Conflict | | -- | -- |  | **-0.22 (0.07)** | **.002** |  | -- | -- |  | **0.19 (0.07)** | **.004** |
|  | | |  |  |  |  |  |  |  |  |  |  |
| ***Interaction Models*** | | |  |  |  |  |  |  |  |  |  |  |
| Testosterone | | -0.11 (0.09) | .22 |  | -0.01 (0.09) | .95 |  | -0.17 (0.09) | .07 |  | -0.09 (0.08) | .30 |
| Pubertal Status | | -0.16 (0.09) | .09 |  | -0.19 (0.10) | .07 |  | **0.22 (0.09)** | **.02** |  | **0.23 (0.09)** | **.01** |
| Testosterone x Pubertal Status | | -0.08 (0.07) | .25 |  | -0.002 (0.07) | .97 |  | -0.07 (0.07) | .31 |  | -0.01 (0.06) | .83 |
| *Covariates:* | | -- | -- |  |  |  |  |  |  |  |  |  |
| Age | | -- | -- |  | -0.06 (0.10) | .55 |  | -- | -- |  | -0.06 (0.09) | .50 |
| BMI | | -- | -- |  | -0.13 (0.07) | .07 |  | -- | -- |  | -0.06 (0.07) | .35 |
| Saliva Collection Time | | -- | -- |  | -0.01 (0.06) | .87 |  | -- | -- |  | -0.10 (0.06) | .09 |
| Fasting Status | | -- | -- |  | -0.19 (0.24) | .42 |  | -- | -- |  | 0.24 (0.22) | .29 |
| Aggressive Behavior | | -- | -- |  | 0.10 (0.07) | .16 |  | -- | -- |  | -- | -- |
| Anxiety Symptoms | | -- | -- |  | -- | -- |  | -- | -- |  | 0.07 (0.07) | .28 |
| Depressive Symptoms | | -- | -- |  | **0.44 (0.08)** | **<.001** |  | -- | -- |  | **0.37 (0.08)** | **<.001** |
| Parent-Child Conflict | | -- | -- |  | **-0.22 (0.07)** | **.003** |  | -- | -- |  | **0.19 (0.07)** | **.004** |
|  | |  |  |  |  |  |  |  | |  |  | |

Note: BMI = Body Mass Index. Continuous variables were standardized prior to analysis so coefficients reflect standardized effects. Fasting status was dummy coded (0 = completed 4 hour fast; 1 = did not fast).

**Table S2.** Post Hoc Analyses Exploring whether Parent-Child Conflict Alters Testosterone and Pubertal Status Effects on Depressive Symptoms in Boys (N = 213).

| ***Effects on Depressive Symptoms*** | Coefficient (S.E.) | *p* |
| --- | --- | --- |
| Testosterone | -0.06 (0.07) | .40 |
| Pubertal Status | 0.13 (0.08) | .14 |
| Parent-Child Conflict | **0.33 (0.07)** | **<.001** |
| Testosterone x Pubertal Status | **-0.13 (0.06)** | **.02** |
| Testosterone x Parent-Child Conflict | 0.05 (0.06) | .69 |
| Pubertal Status x Parent-Child Conflict | 0.07 (0.07) | .34 |
| Testosterone x Pubertal Status x Parent-Child Conflict | 0.05 (0.05) | .33 |
|  |  |  |
| *Covariates:* |  |  |
| Age | 0.01 (0.08) | .91 |
| BMI | **0.14 (0.06)** | **.02** |
| Saliva Collection Time | -0.03 (0.05) | .61 |
| Fasting Status | 0.22 (0.19) | .26 |
| Anxiety Symptoms | **0.30 (0.06)** | **<.001** |
| Aggressive Behavior | **0.26 (0.06)** | **<.001** |

Note: BMI = Body Mass Index. Continuous variables were standardized prior to analysis so coefficients reflect standardized effects. Fasting status was dummy coded (0 = completed 4 hour fast; 1 = did not fast).


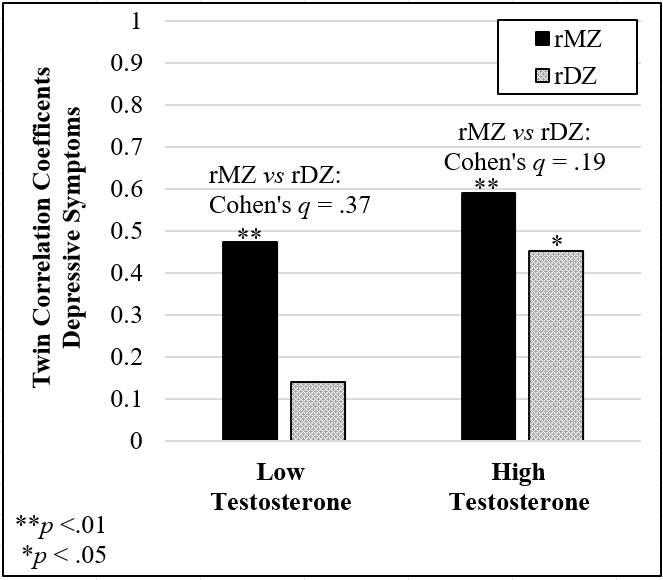


**Fig S1.** Post hoc monozygotic (MZ) and dizygotic (DZ) twin correlations for depressive symptoms by low and high testosterone levels. The difference in genetic relatedness of MZ twins (who share 100% of their segregating genes) and DZ twins (who share, on average, 50% of their segregating genes) can be used to explore genetic and environmental influences on outcomes, like depressive symptoms. If the MZ twin correlation is at least twice as similar as the DZ twin correlation, this indicates the presence of genetic effects. If the MZ and DZ twin correlations are greater than 0 and are similar in magnitude, this reflects the presence of shared environmental influences (i.e., environmental factors that are common to siblings growing up in the same family and contribute to their behavioral similarity) and a lack of genetic effects. When MZ twin correlations are less than 1.00, this indicates the presence of nonshared environmental factors (i.e., factors that are unique to siblings growing up in the same family and contribute to behavioral differences) as well as measurement error. For this exploratory analysis, we divided same-sex male twins into low versus high testosterone groups using the median split for testosterone levels (41.18 pg/mL), and depressive symptom scores were adjusted for age, pubertal status, and BMI to ensure these potentially confounding variables did not unduly impact results. Because twin intra-class correlations require the inclusion of both twins from a twin pair, only same-sex male twin pairs that had a co-twin concordant on testosterone status could be included (N = 116 twins, or 58 twin pairs; 54.5% of total sample). We confirmed, however, that there were no significant mean or variance differences in depressive symptoms (*p*s = .45-.55) between the subset of twins included in these analyses (i.e., same-sex male twins concordant on testosterone status) versus those excluded (i.e., males from opposite-sex twin pairs or same-sex male twins discordant on testosterone status). Cohen’s *q* represents the effect size estimate for the difference between the rMZ and rDZ twin correlations in each testosterone group, which can be interpreted in terms of a small (.1 to .3), intermediate (.3 to .5), or large (>.5) effect.

**References**

Burt, S.A., Clark, D.A., Gershoff, E.T., Klump, K.L., & Hyde, L.W. (2021). Twin differences in

harsh parenting predict youth’s antisocial behavior. *Psychological science*, *32*, 395-409.

Capaldi, D.M. & Rothbart, M.K. (1992).  Development and Validation of an Early Adolescent

Temperament Measure. *Journal of Early Adolescence, 12*, 153-173.

Elkins, I.J., McGue, M., & Iacono, W.G. (1997) Genetic and environmental influences on parent–

son relationships: Evidence for increasing genetic influence during adolescence.

*Developmental Psychology, 33*, 351-363.

Ellis, L. K., & Rothbart, M. K. (2001). Revision of the Early Adolescent Temperament

Questionnaire. Poster presented at the Biennial Meeting of the Society for Research in Child

Development. Minneapolis, Minnesota.

Grills-Taquechel, A.E., Ollendick, T.H., & Fisak B. (2008). Reexamination of the MASC factor

structure and discriminant ability in a mixed clinical outpatient sample. *Depression and*

*Anxiety, 25,* 942–950.

Klahr, A.M., Rueter, M.A., McGue, M., Iacono, W.G., & Burt, S.A. (2011). The

relationship between parent-child conflict and adolescent antisocial behavior: Confirming

shared environmental mediation. *Journal of Abnormal Child Psychology*, *39*, 683-694.

March, J.S., Parker, J., Sullivan, K., Stallings, P., &Conners, C.K. (1997). The Multidimensional

Anxiety Scale for Children (MASC): factor structure, reliability, and validity. *Journal of the*

*American Academy of Child and Adolescent Psychiatry, 36*, 554–565.

March, J.S., Sullivan, K., & Parker, J. (1999). Test–retest reliability of the multidimensional anxiety

scale for children. *Journal of Anxiety Disorders,* 13, 349–358.

Wood, J.J., Piacentini, J.C., Bergman, R.L., McCracken, J., Barrios, V. (2002). Concurrent validity

of the anxiety disorders section of the Anxiety Disorders Interview Schedule for DSM-IV: Child and parent versions. *Journal of Clinical Child & Adolescent Psychology, 31*, 335–342.
